# Renal effects of Sacubitril/Valsartan in heart failure with reduced ejection fraction: a real life 1-year follow-up study

**DOI:** 10.1007/s11739-019-02111-6

**Published:** 2019-05-30

**Authors:** Francesco Spannella, Marco Marini, Federico Giulietti, Giulia Rosettani, Matteo Francioni, Gian Piero Perna, Riccardo Sarzani

**Affiliations:** 1 Internal Medicine and Geriatrics, IRCCS-INRCA, Ancona, Italy; 2 Department of Clinical and Molecular Sciences, University “Politecnica Delle Marche”, Ancona, Italy; 3 Department of Cardiovascular Sciences, Ospedali Riuniti, Ancona, Italy; 4 Internal Medicine and Geriatrics, Department of Clinical and Molecular Sciences, University “Politecnica Delle Marche”, Italian National Research Centre on Aging, Hospital “U. Sestilli”, IRCCS-INRCA, Via Della Montagnola n. 81, 60127 Ancona, Italy

**Keywords:** Sacubitril/Valsartan, Heart failure, Renal function, Blood pressure

## Abstract

Real-life data confirming the favourable renal outcome in patients with heart failure (HF) treated with Sacubitril/Valsartan, previously found in several trials (RCTs), are still scant. We evaluated the renal effects of Sacubitril/Valsartan in a real-life sample of HF patients. Observational analysis of 54 consecutive outpatients affected by HF with reduced ejection fraction (HFrEF) and clinical indication for Sacubitril/Valsartan. Patients were evaluated at baseline (T0) and after six (T6) and twelve (T12) months after initiating Sacubitril/Valsartan and compared with a group of 30 historical controls. Mean age: 65.5 ± 11.7 years. Older patients: 29 (53.7%). Mean baseline estimated glomerular filtration rate (eGFR): 59.4 ± 19.2 ml/min/1.73 m^2^. Patients with chronic kidney disease (CKD), defined by an eGFR < 60 ml/min/1.73 m^2^, were 29 (53.7%). Sacubitril/Valsartan was less titrated in both older patients and patients with CKD. There were no changes in diuretics during follow-up. Systolic blood pressure (BP) decreased during follow-up (*p* = 0.014), while left ventricular ejection fraction (LVEF) slighly increased (*p* < 0.001). Renal function improved after 12 months compared to historical controls (*p* for interaction < 0.001) and a greater benefit was found in subjects aged < 65 years (*p* for interaction = 0.002) and patients with CKD (*p* for interaction = 0.009). A statistically (*p* = 0.009), but not clinically significant increase in serum potassium was also found, regardless of age and CKD. This is the first study focused on the renal effects of Sacubitril/Valsartan in HFrEF patients followed for 12 months in a real-life clinical context. The improved eGFR, despite lower BP, represents an important confirmation outside the peculiar world of RCTs.

## Introduction

Heart failure (HF) is a major global health concern, given its estimated prevalence of nearly 62 million patients worldwide [1]. This prevalence is approximately 1%–2% of the adult population in developed countries, rising to over 10% among people aged 70 years or older [2]. To date, the inhibition of the renin–angiotensin–aldosterone system (RAAS) and the sympathetic nervous system (SNS) by angiotensin-converting-enzyme inhibitors or angiotensin receptor blockers (ACE-I/ARBs) and beta-blockers (BBs) has been the cornerstone of drug therapy for HF with reduced ejection fraction (HFrEF) [3]. Despite these therapies, more than half of HFrEF patients continue to die within 5 years [4]. Therefore, medical research has sought to identify new therapeutic targets to improve these outcomes.

Cardiac natriuretic peptides (NPs) release is stimulated by cardiac muscular wall stretch, resulting from increased intravascular volume and/or transmural pressure, and a dysregulation of the NPs system has been found in HF patients [5]. NPs reduce renal and systemic vascular resistances and promote natriuresis and diuresis. Therefore, in patients with HFrEF, NPs play a key role in maintaining sodium and fluid balance, despite the hyperactivation of the RAAS typically found in such patients [6]. In the PARADIGM-HF trial, the first-in-class angiotensin receptor-neprilysin inhibitor Sacubitril/Valsartan, that combines the benefits derived from the inhibition of both the RAAS and the degradation of cardiac NPs, was found to reduce the risk of cardiovascular (CV) death and hospitalization due to HFrEF by 20%, compared to the standard of care (Enalapril), with lower proportion of renal impairment and hyperkalemia [7], and a projected increase in life expectancy and survival free from HF of 1–2 years [8].

Chronic HF and chronic kidney disease (CKD) frequently coexist, due to the shared risk factors (i.e. hypertension, diabetes, hyperlipidaemia) and are associated with a sharp increase of mortality risk [9, 10]. Patients with HFrEF experience a faster decline in renal function compared with general population [11], due to reduced blood pressure (BP), renal perfusion and GFR. Following initiation and uptitration of RAAS inhibitors in HFrEF patients, a worsening renal function is relatively common, although usually small, and should not lead to treatment discontinuation [12, 13]. Sacubitril/Valsartan, by increasing endogenous NPs levels through the inhibition of neprilysin and simultaneously blocking the RAAS, exerts beneficial effects on cardio-renal system [14]. Indeed, data from clinical trials (RCTs) showed better eGFR progression in HF patients treated with Sacubitril/Valsartan compared to RAAS inhibitors alone [7, 15].

To date, focused evidence on the renal effects of Sacubitril/Valsartan in patients with HFrEF from real-life clinical practice is still lacking in the published literature. The aim of our study was to evaluate the effects of Sacubitril/Valsartan on the renal function in real-life consecutive HFrEF patients. We hypothesized that Sacubitril/Valsartan might have a beneficial role on eGFR, as shown in several RCTs.

## Methods

### Study design and population

We performed a longitudinal, observational, two-center study on 60 consecutive HFrEF outpatients referred to the Department of Cardiovascular Sciences, Ospedali Riuniti (Ancona, Italy) or to Internal Medicine and Geriatrics, IRCCS-INRCA (Ancona, Italy) between October 2016 and October 2017. We considered the following inclusion criteria: age ≥ 18 years and a diagnosis of HFrEF with clinical indication for Sacubitril/Valsartan [16]. We only included patients being initiated on Sacubitril/Valsartan as ambulatory patients with chronic HFrEF. We excluded patients with dementia and conditions with a life expectancy of less than one year (due to conditions such as end-stage renal disease, decompensated cirrhosis or advanced cancer). In the analyses, we also excluded patients (*n* = 4) who had medical or surgical diseases determining a significant impairment of renal function during the follow-up (i.e. nephrological/urological diseases). These conditions, unrelated to the study drug, could have biased our results, given that the aim of our study was to evaluate the trend of renal function in stable chronic conditions. Furthermore, two patients were lost to follow-up, while no patients died during the study phases. Therefore, statistical analyses were conducted on 54 patients who completed the follow-up successfully.

Patients eligible for Sacubitril/Valsartan were followed with medical evaluations at baseline (T0), after 6 months (T6) and after 12 months (T12). At recruitment, all patients were taking an optimal medical therapy for HFrEF. Sacubitril/Valsartan was administered according to the Italian reimbursement criteria: chronic symptomatic HF defined as New York Heart Association (NYHA) class II–III symptoms, left ventricular ejection fraction (LVEF) ≤ 35% measured by echocardiography, pre-treatment with a maximally tolerated dose of ACE-I or ARBs. During the follow-up, all patients were treated according to the “good clinical practice” (GCP). In particular, Sacubitril/Valsartan was titrated, whenever possible, and the dosages of other drugs for HF were modulated according to common clinical parameters (symptoms/signs, BP, electrocardiography and laboratory parameters). Informed consent was obtained from all individual participants included in the study. Designing a controlled study with similar patients not treated with Sacubitril/Valsartan would have raised important ethical concerns. Therefore, we used a group of historical patients, evaluated between October 2015 and October 2016 for HFrEF, to compare the eGFR trend between the two groups. Thirty historical controls were consecutively recruited according to the same inclusion/exclusion criteria of the study population, followed at the same time intervals, and received optimal medical therapy, except for the administration of Sacubitril/Valsartan, that was not yet available.

### Clinical parameters

At baseline and during follow-up clinical visits, we evaluated demographics, physical features, BP and heart rate values, aetiology of HF, presence of comorbidities, NYHA-class, laboratory and transthoracic echocardiogram features [LVEF, diameter of the inferior vena cava, estimated systolic pulmonary artery pressure (PAPs)], and drug therapy. In particular, LVEF was evaluated with transthoracic 2D-echocardiography using the biplane method of disks (modified Simpson method). The echocardiographic evaluations throughout the study phases were performed by the same cardiologist, following a standardised protocol, to minimize intra-observer bias and avoid inter-observer bias. Furthermore, the echocardiography was performed the same day of the evaluation of all the other parameters. During the clinical visits, we performed three sequential oscillometric automatic BP measurements (using Microlife® BP A200 AFib, Widnau, Switzerland), considering the same arm in the follow-up visits. Correct cuff sizes (range 22–32 cm or 32–42 cm) were selected according to arm circumference and BP measurements were performed after at least 5 min of rest in the sitting position. The patient’s arm was kept at the heart level during the measurement. Smoking status was ascertained during recruitment and smoking habit was defined as current smoking or previous smoking of at least 100 cigarettes in a lifetime [17]. We considered the following laboratory parameters: serum creatinine, eGFR, serum sodium, serum potassium, NT-proBNP, hemoglobin. Renal function was assessed by serum creatinine and eGFR, which was calculated using the CKD-EPI creatinine equation [18]. Regarding patients who experienced an acute HF exacerbation during follow-up, we took into account the renal function after stabilization of the acute phase. CKD was defined as an eGFR < 60 ml/min/1.73 m^2^ in at least two previous creatinine determinations, obtained at least three months apart from each other, before starting Sacubitril/Valsartan.

In addition to Sacubitril/Valsartan, the following CV drug classes were also considered: ACE-I and ARBs, BBs, mineralocorticoid receptor antagonists (MRA), loop diuretics, ivabradine, digoxin, statins, antiplatelet and antithrombotic agents. To compare the dosages of different drugs within the same drug class and their changes over time, as previously reported [19], the daily dose taken by the patient was divided by the maximum recommended daily dose to obtain a proportional dose [called treatment intensity score (TIS)] for that medication. For example, a patient taking an 80-mg daily dose of a drug for which 160 mg is the “maximum daily dose” recommended was considered to be taking 0.5 intensity units. For completeness, dual-class drugs were separated into their components, and TIS was calculated separately for each chemical compound. The maximum recommended daily doses, based on the target dose set by the 2016 ESC Guidelines for the diagnosis and treatment of acute and chronic HF [16], were used for calculations. Given the difficulty of establishing the maximum dosage and the wide range of dosages taken by patients, treatment intensity of loop diuretics (furosemide was the only diuretic took by all patients) was considered on the basis of daily dose (mg/day).

### Statistical analysis

Statistical analysis was performed with the SPSS software (Version 13 for Windows; SPSS, Inc., Chicago, IL). Data were expressed as mean ± standard deviation for normal distribution variables (except where otherwise specifically provided) and with median/interquartile range for non-normal distributions. A *p *value < 0.05 was considered statistically significant. Repeated measures analysis of variance (ANOVA), repeated measures analysis of co-variance (ANCOVA), McNemar test and Friedman test (for non-normal distributions) were used to assess the differences of the selected variables at the specified time-intervals T0, T6, T12. Unpaired *t *test and Chi-square analysis were used to evaluate differences between subgroups considered.

## Results

General characteristics of both study population (*n* = 54 patients), before starting therapy with Sacubitril/Valsartan, and historical controls (*n* = 30 patients) are summarized in Table 1. Regarding study population, mean age was 65.5 ± 11.7 years (range of age 44–95 years), with male prevalence. Older patients (age ≥ 65 years) were 29 (53.7%), patients with overweight/obesity were 35 (64.8%) and patients with CKD were 29 (53.7%). The main causes of HF were ischemic heart disease and dilated cardiomyopathy. At baseline, 37.0% and 63.0% of patients had NYHA class II and class III symptoms, respectively. Symptoms improved during the follow-up (prevalence of NYHA class III decreased from 63.0% at baseline to 13.0% at T6 and 14.8% at T12). Seven patients out of 54 (13.0%) experienced an acute HF exacerbation during follow-up. All studied patients took an ACE-I or an ARB, nearly all patients took a BB and a loop diuretic, more than 70% of patients took a MRA.

**Table 1:** General characteristics of both study population, before starting therapy with Sacubitril/Valsartan, and historical controls

Clinical characteristics	Study population (*n* = 54)	Historical controls (*n* = 30)	*p*
Age (years)	65.5 ± 11.7	65.1 ± 10.7	0.899
Sex (male)	40 (74.1%)	17 (56.7%)	0.102
BMI (kg/m^2^)	27.1 ± 4.9	25.0 ± 3.2	0.021
Etiology of heart failure			
Ischemic heart disease	32 (59.3%)	26 (86.7%)	–
Dilated cardiomyopathy	18 (33.3%)	4 (13.3%)	–
Inflammatory cardiomyopathy	2 (3.7%)	–	–
Valvular heart disease	1 (1.9%)	–	–
Chemotherapy-induced cardiomyopathy	1 (1.9%)	–	–
LVEF (%)	29.7 ± 4.9	30.3 ± 4.3	0.566
Hypertension	39 (72.2%)	27 (90.0%)	0.057
Dyslipidemia	40 (74.1%)	28 (93.3%)	0.031
Diabetes mellitus	11 (20.4%)	11 (36.7%)	0.104
Smoking	24 (44.4%)	16 (53.3%)	0.434
Coronary artery disease	32 (59.3%)	26 (86.7%)	0.014
Atrial fibrillation	24 (44.4%)	6 (20.0%)	0.025
Chronic obstructive pulmonary disease	12 (22.2%)	8 (26.7%)	0.647
Systolic blood pressure (mmHg)	119.0 ± 14.3	118.3 ± 12.4	0.778
Diastolic blood pressure (mmHg)	72.2 ± 10.1	69.0 ± 7.7	0.107
Heart rate (bpm)	68 (60–80)	65 (60–70)	0.145
Drug/device therapy			
ACE-I	30 (55.6%)	11 (36.7%)	0.596
ARBs	24 (44.4%)	19 (63.3%)	0.068
Beta blocker	49 (90.7%)	30 (100%)	0.155
MRA	40 (74.1%)	23 (76.7%)	0.793
Diuretic	49 (90.7%)	29 (96.7%)	0.414
Ivabradine	8 (14.8%)	2 (6.7%)	0.483
Digoxin	14 (25.9%)	5 (16.7%)	0.331
Warfarin	13 (24.1%)	6 (20.0%)	0.669
DOAC	10 (18.5%)	3 (10.0%)	0.362
Antiplatelet therapy	25 (46.3%)	21 (70.0%)	0.036
Statin	35 (64.8%)	28 (93.3%)	0.004
CRT/ICD	38 (70.4%)	19 (63.3%)	0.508
Laboratory parameters			
Creatinine (mg/dl)	1.28 ± 0.34	1.16 ± 0.32	0.131
eGFR (ml/min/1.73 m^2^)	59.4 ± 19.2	63.0 ± 19.1	0.412
Sodium (mmol/l)	140.0 ± 2.8	139.5 ± 3.0	0.441
Potassium (mmol/l)	4.2 ± 0.4	4.1 ± 0.3	0.033
NT-proBNP (pg/ml)	1736 (1366–4323)	3808 (1820–4840)	0.364
Hemoglobin (g/dl)	13.0 ± 1.5	12.7 ± 1.0	0.248

All continuous variables were expressed as mean ± SD, except heart rate that was expressed as median and interquartile range, because markedly skewed. Categorical variables were expressed as absolute number and percentage

*BMI* body mass index, *LVEF* left ventricular ejection fraction, *ACE-I* angiotensin converting enzyme inhibitor, *ARBs* angiotensin receptor blockers, *MRA* mineralocorticoid receptor antagonists, *DOAC* direct oral anticoagulant, *CRT* cardiac resynchronization therapy, *ICD* implantable cardioverter defibrillator, *eGFR* estimated glomerular filtration rate, *NT-proBNP* N-terminal-proB-type natriuretic peptide

### Changes in drug therapy, blood pressure and echocardiographic parameters

The rates of prescription of HF drugs throughout the study phases are illustrated in Table 2. Sacubitril/Valsartan was progressively titrated, starting from the lowest dose in the most of patients, and no patients discontinued Sacubitril/Valsartan during the follow-up. The dosage of BBs increased [TIS 0.250 (0.250–0.500) at baseline vs 0.375 (0.250–0.500) at T6 vs 0.500 (0.250–0.500) at T12, *p* = 0.001], while there were no changes in the MRA [TIS 0.500 (0.250–0.500) in all the time intervals considered, *p* = 0.882] in the study population during follow-up. No significant changes in dosages [50 (25–75) mg at baseline vs 50 (25–87) mg at T6 vs 50 (25–75) mg at T12, *p* = 0.299] of loop diuretics were found during the study phases.

**Table 2:** Prescription rates of HF drugs during the study phases

	Study population (*n* = 54 patients)			Historical controls (*n* = 30 patients)		
	T0	T6	T12	T0	T6	T12
Sacubitril/Valsartan 24/26 mg	47 (87%)	7 (13%)	0 (0%)	–	–	–
Sacubitril/Valsartan 49/51 mg	25 (46.3%)	22 (40.7%)	7 (13%)	–	–	–
Sacubitril/Valsartan 97/103 mg	20 (37.0%)	21 (38.9%)	13 (24.1%)	–	–	–
ACE-I	–	–	–	11 (36.7%)	11 (36.7%)	11 (36.7%)
ARBs	–	–	–	19 (63.3%)	19 (63.3%)	19 (63.3%)
Beta blockers	53 (98.1%)	51 (94.4%)	53 (98.1%)	30 (100%)	30 (100%)	30 (100%)
MRA	36 (66.7%)	38 (70.4%)	36 (66.7%)	24 (80%)	24 (80%)	25 (83.3%)
Diuretics	47 (87.0%)	44 (81.5%)	46 (85.2%)	29 (96.7%)	29 (96.7%)	29 (96.7%)
Ivabradine	11 (20.4%)	10 (18.5%)	11 (20.4%)	2 (6.7%)	2 (6.7%)	2 (6.7%)
Digoxin	16 (29.6%)	12 (22.2%)	14 (25.9%)	6 (20%)	6 (20%)	6 (20%)

All variables were expressed as absolute number and percentage. All *p* between time intervals (Reference: T0) > 0.05

*ACE-I* angiotensin-converting-enzyme inhibitors, *ARBs* angiotensin receptor blockers, *MRA* mineralocorticoid receptor antagonists

Regarding the historical controls, ACE-I/ARBs were titrated over time [TIS 0.250 (0.125–0.500) at baseline vs 0.500 (0.250–0.500) at T6 vs 0.500 (0.250–0.500) at T12, *p* < 0.001], as well as BB [TIS 0.250 (0.125–0.313) at baseline vs 0.250 (0.250–0.500) at T6 vs 0.375 (0.250–0.500) at T12, *p* < 0.001] and MRA [TIS 0.250 (0.250–0.250) at baseline vs 0.500 (0.250–0.500) at T6 vs 0.500 (0.250–0.500) at T12, *p* = 0.014], while there were no changes in the dosages of loop diuretics [50 (38–75) mg at baseline vs 50 (25–100) mg at T6 vs 50 (25–100) mg at T12, *p* = 0.607].

The trends of systolic BP, diastolic BP and echocardiographic parameters in both study population and historical controls are described in Table 3. In the study population, both systolic and diastolic BP significantly decreased during follow-up, although no symptomatic hypotension was reported. We also found a statistically significant improvement in LVEF, but no clinical class changes. On the other hand, we found no significant changes in both diameter of the inferior vena cava and PAPs (Table 3).

**Table 3:** Changes in blood pressure and echocardiographic parameters during the study phases

	Study population (*n* = 54 patients)				Historical controls (*n* = 30 patients)			
	T0	T6	T12	*p*	T0	T6	T12	*p*
Systolic BP (mmHg)	119.0 ± 14.3	113.3 ± 15.5	114.8 ± 15.8	0.014	118.3 ± 12.4	116.2 ± 12.7	114.7 ± 8.4	0.145
Diastolic BP (mmHg)	72.2 ± 10.1	67.3 ± 11.1	67.5 ± 10.0	0.002	69.0 ± 7.7	67.8 ± 7.7	67.0 ± 6.2	0.458
LVEF (%)	29.7 ± 5.0	32.7 ± 5.6	32.2 ± 7.2	< 0.001	30.3 ± 4.3	30.8 ± 4.0	31.3 ± 3.1	0.004
IVC diameter (mm)	17.7 ± 2.4	17.4 ± 1.7	17.8 ± 2.2	0.443	17.9 ± 3.0	17.8 ± 2.6	17.9 ± 3.6	0.878
PAPs (mmHg)	37.0 ± 8.8	37.5 ± 12.0	36.6 ± 11.9	0.864	40.1 ± 11.0	38.8 ± 11.4	36.8 ± 10.8	0.294

*BP* blood pressure, *LVEF* left ventricular ejection fraction, *IVC* inferior vena cava, *PAPs* estimated systolic pulmonary artery pressure

### Changes in renal function

**Fig.1:**
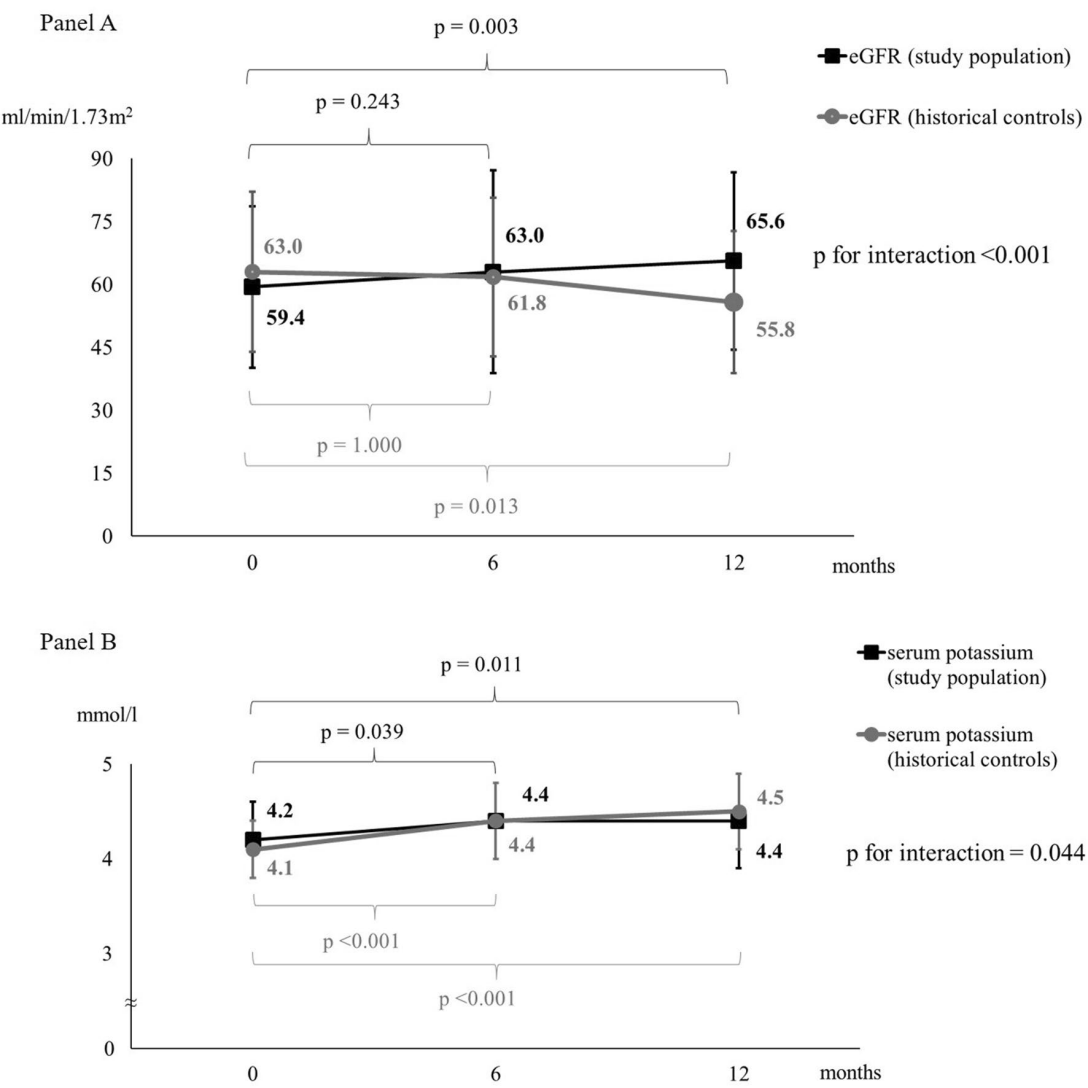
Changes in renal function in both study population (*n* = 54 patients) and historical controls (*n* = 30 patients). Panel **a** Changes in estimated glomerular filtration rate (eGFR). Panel **b** Changes in serum potassium

Renal function significantly improved after 12 months in the study population compared to historical controls, as described in Fig. 1, Panel A. This finding remained statistically significant even after adjustments for age and sex (*p* < 0.001). In the study population, there was no interaction between eGFR trend and systolic BP or LVEF at baseline (*p* for interaction = 0.479 and p for interaction = 0.432, respectively). Furthermore, no significant difference in eGFR trend was observed between patients who experienced an acute HF exacerbation during follow-up and patients who did not (*p* for interaction = 0.997) and between patients with baseline NTproBNP below or above the median (*p* for interaction = 0.431). Serum potassium significantly increased in the study population, but not in a clinically significant manner, as well as in the historical controls (see Fig. 1, Panel B). No severe hyperkalemia (serum potassium levels ≥ 5.5 mmol/l) was found at T6, while only one case was found at T12 (serum potassium = 5.9 mmol/l), despite over 74% of studied patients were also treated with MRA. Serum sodium did not change significantly in study population during the follow-up (140.0 ± 2.8 mmol/l at baseline vs 139.8 ± 3.6 mmol/l at T6 vs 140.4 ± 4.1 mmol/l at T12, *p* = 0.616).

### Changes in renal function according to age and chronic kidney disease in the study population

**Fig.2:**
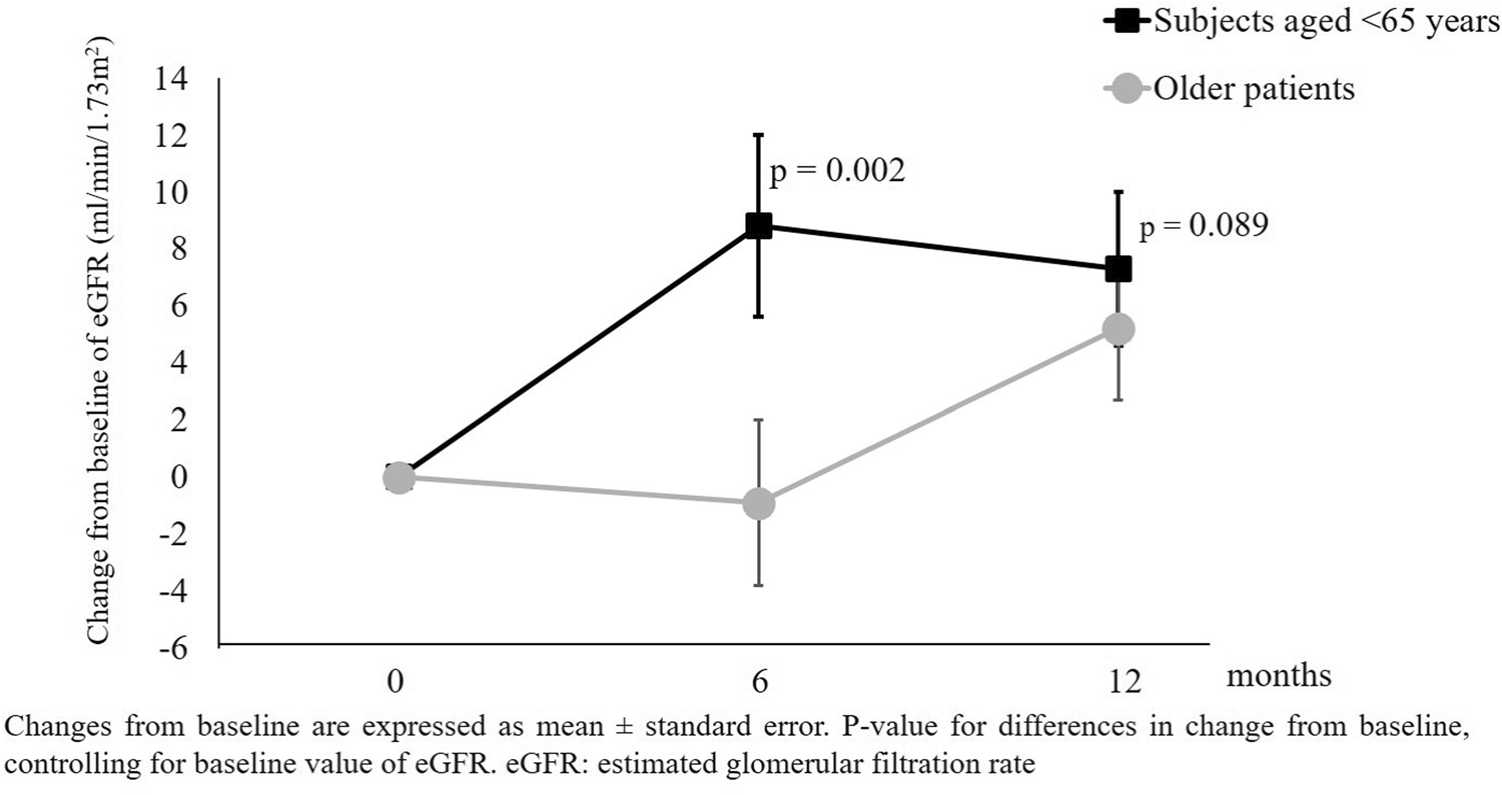
Changes in estimated glomerular filtration rate (eGFR) according to age in the study population (*n* = 54 patients)

We specifically focused the analysis on the effects of Sacubitril/Valsartan on renal function in older patients and patients with CKD. Older patients had higher prevalence of CKD (72.4% vs 32.0%, *p* = 0.003), dyslipidemia (86.2% vs 60.0%, *p* = 0.028), atrial fibrillation (65.5% vs 20.0%, *p* = 0.001) and chronic obstructive pulmonary disease (34.5% vs 8.0%, *p* = 0.020). There was no difference in sex (*p* = 0.117). They had a lower baseline eGFR than subjects aged < 65 years (49.5 ± 14.9 vs 70.9 ± 17.2 ml/min/1.73 m^2^, *p* < 0.001). In older patients, Sacubitril/Valsartan was less titrated compared to subjects aged < 65 years (see Table 4). There was a different trend in renal function between subjects aged < 65 years and older patients (*p* for interaction = 0.002), as shown in Fig. 2. Subjects aged < 65 years experienced a greater improvement in eGFR compared to older patients. On the other hand, the trends of systolic BP (*p* for interaction = 0.425), LVEF (*p* for interaction = 0.952) and serum potassium (*p* for interaction = 0.565) did not differ between subjects aged < 65 years and older patients.

**Table 4:** Titration of Sacubitril/Valsartan in older patients and patients with CKD (n = 54 patients)

Study phase	Drug dosages	Age < 65 (*n* = 25) (%)	Age ≥ 65 (*n* = 29) (%)	*p*	eGFR < 60 ml/min/1.73 m^2^ (*n* = 29) (%)	eGFR ≥ 60 ml/min/1.73 m^2^ (*n* = 25) (%)	*p*
T0	Sacubitril/Valsartan 24/26 mg	80.0	93.1	0.153	84.0	89.7	0.537
	Sacubitril/Valsartan 49/51 mg	20.0	6.9		16.0	10.3	
	Sacubitril/Valsartan 97/103 mg	0	0		0	0	
T6	Sacubitril/Valsartan 24/26 mg	28.0	62.1	0.037	28.0	62.1	0.042
	Sacubitril/Valsartan 49/51 mg	52.0	31.0		56.0	27.6	
	Sacubitril/Valsartan 97/103 mg	20.0	6.9		16.0	10.3	
T12	Sacubitril/Valsartan 24/26 mg	24.0	48.3	0.006	32.0	41.4	0.036
	Sacubitril/Valsartan 49/51 mg	32.0	44.8		28.0	48.3	
	Sacubitril/Valsartan 97/103 mg	44.0	6.9		40.0	10.3	

*eGFR* estimated glomerular filtration rate

**Fig.3:**
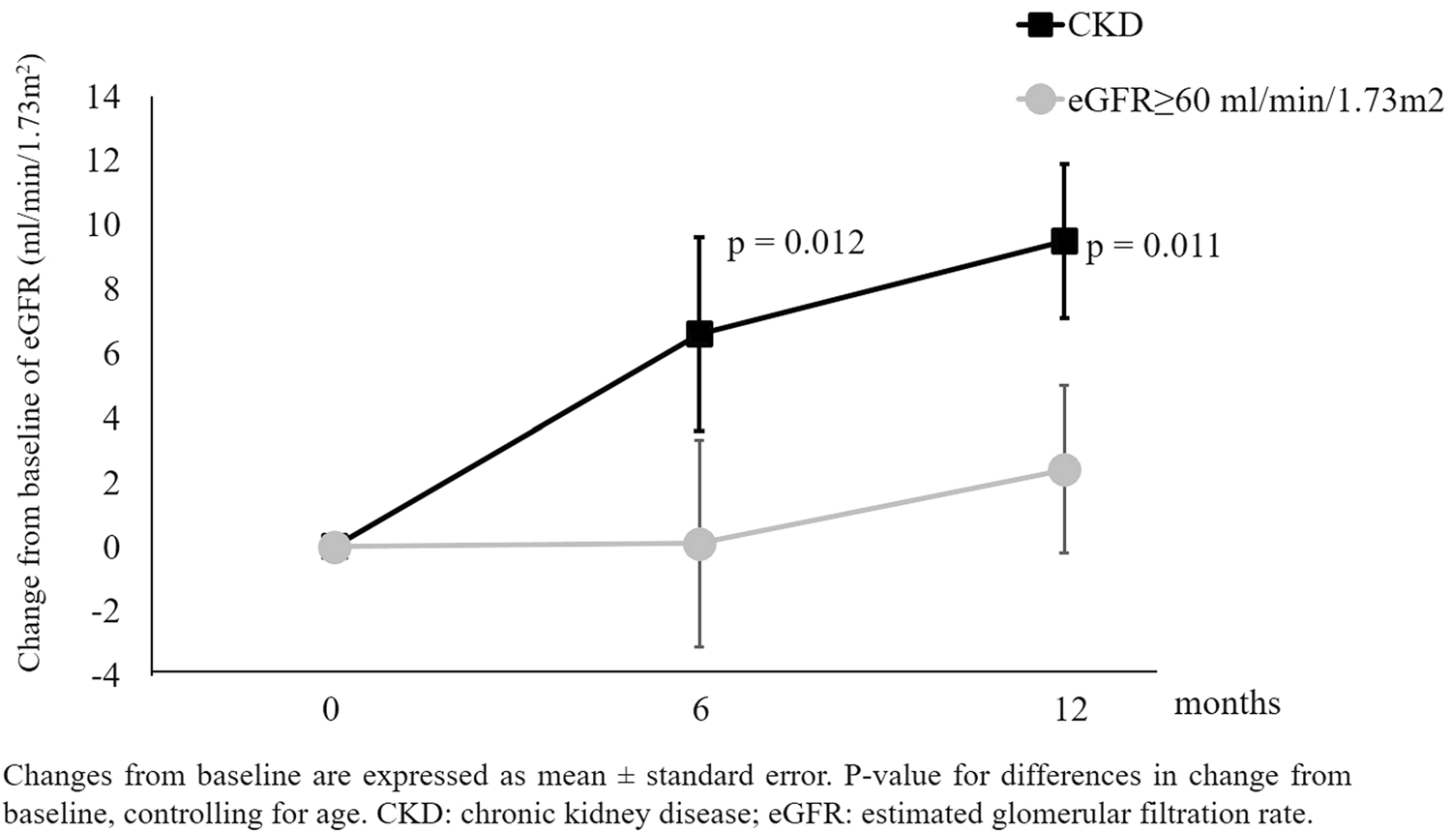
Changes in estimated glomerular filtration rate (eGFR) according to CKD in the study population (*n* = 54 patients)

Patients with CKD had higher prevalence of atrial fibrillation (58.6% vs 28.0%, *p* = 0.024) and diabetes mellitus (31.0% vs 8.0%, *p* = 0.036). There was no difference in sex (*p* = 0.122). Sacubitril/Valsartan was less titrated in patients with CKD compared to patients with eGFR ≥ 60 ml/min/1.73 m^2^ (see Table 4). Moreover, patients with CKD took higher dosages of loop diuretics [25 (25–69) mg vs. 50 (25–100) mg, *p* = 0.029]. Figure 3 shows the changes in eGFR according to presence/absence of CKD (p for interaction adjusted for age = 0.009). Patients with CKD at baseline had a greater beneficial at T6 and T12. The trends of systolic BP (*p* for interaction = 0.349), LVEF (*p* for interaction = 0.433) and serum potassium (*p* for interaction = 0.564) did not differ between patients with CKD and those with eGFR ≥ 60 ml/min/1.73 m^2^.

## Discussion

In our real-life clinical study on patients with HFrEF, Sacubitril/Valsartan improved eGFR, despite a decrease in BP values, and no clinical increase in serum potassium was observed. Subjects aged < 65 years and patients with CKD were those who showed a greater benefit. Our study confirmed the positive findings of dual RAAS-neprilysin inhibition on renal function showed in previous RCTs [15, 20] and added further detailed information on elderly and CKD patients.

The problem of the generalizability of RCTs results in real-world patients is well known. Clinical trials use strict inclusion and exclusion criteria and real-world patients rarely fit into those tight frames. The real-world HF population is generally older and suffer from more comorbidities [3]. Patients in real-life clinical practice have a higher risk of hospitalization and death, and often are not able to tolerate the high dosages achieved in the RCTs [21–23].

In several RCTs on HF patients, Sacubitril/Valsartan was found to improve creatinine and eGFR. On the other hand, an increase in urinary albumin-to-creatinine ratio (UACR) was also reported, compared to patients treated with ACE-I alone [15]. The beneficial effect on eGFR occurred despite the significant BP reduction that usually leads to a decrease in eGFR in HF patients, especially during treatment with RAAS blockers. In the PARADIGM-HF trial, the rate of worsening renal function was lower in the arm treated with Sacubitril/Valsartan compared to the arm treated with Enalapril [7]. Furthermore, in a post-hoc analysis, where the study population was divided in prespecified sub-groups (patients with and without CKD, based on eGFR < 60 ml/min/1.73 m^2^), Damman et al. found that Sacubitril/Valsartan had a favorable effect on renal outcomes, regardless of the presence of CKD [24]. Moreover, the magnitude of the benefit of neprilysin inhibition on renal function was found to be significantly greater in diabetic patients [25], in whom a degradation of endogenous NPs through an increased activity of neprilysin in all target tissues affected by vascular disease has been reported [26, 27]. In this context, kidney function may be particularly affected by the reduced protective effects of NPs [28], and the pharmacological inhibition of neprilysin could slow down, or even improve the diabetic kidney disease [29].

In our sample, we found that patients with CKD showed a greater improvement in eGFR compared to patients with eGFR ≥ 60 ml/min/1.73 m^2^, although they were taking higher dosages of loop diuretics and lower dosages of Sacubitril/Valsartan.

In the PARAMOUNT trial, Voors et al. found similar results in patients with HFpEF [15]. In particular, patients treated with Sacubitril/Valsartan showed lower serum creatinine and higher eGFR after 36 weeks of treatment and also greater BP reduction and higher UACR, compared to patients treated with Valsartan alone. Investigators speculated that this finding could have led to lower rates of discontinuation or underdosing in clinical practice and a positive prognostic role in patients with HFpEF. On the other hand, they highlighted the uncertainty about the prognostic significance of the rise in UACR, stating that it might not be a marker of disease progression, but an intrinsic effect of the drug [15].

In our study population, older patients had a worse baseline renal function and a different eGFR progression, compared to subjects aged < 65 years. Indeed, older patients showed a delayed benefit that only occurred after 12 months of therapy. This finding was not due to hemodynamic effects, since BP and LVEF did not differ between the two subgroups. We speculated that this could be due to the lower dosages of Sacubitril/Valsartan taken by older patients and to the greater baseline renal damage with a greater glomerulosclerosis [30] and a consequent greater latency in manifesting a change in the eGFR. The PARADIGM-HF trial recruited 4120 patients aged ≥ 65 years and age had no significant impact on patients’ primary outcomes. Moreover, renal function and serum potassium were less affected in the Sacubitril/Valsartan arm, independently from the age of participants [31].

The UK Heart And Renal Protection III (UK HARP-III) trial was the first RCT that specifically addressed the effects of Sacubitril/Valsartan on kidney function [32]. It recruited 414 patients with CKD, defined as eGFR ≥ 45 and < 60 ml/min/1.73 m^2^ with UACR > 20 mg/mmol or eGFR between 20 and 45 ml/min/1.73 m^2^. Twelve-month treatment with Sacubitril/Valsartan compared with Irbesartan did not significantly affect kidney function in people with CKD and was well tolerated. Sacubitril/Valsartan had no additional effect on albuminuria in this population [32]. Therefore, this trial on CKD patients did not confirm findings from previous studies on HF patients that had indicated a better renal outcome with Sacubitril/Valsartan compared to a RAAS inhibitor alone [15, 25]. Different determinants of kidney disease progression between CKD patients and HF patients may explain these findings. Indeed, half of participants had causes of CKD not mediated by glomerulosclerosis, and therefore, the progression of the disease could not be modifiable by drug therapy [33]. However, the UK HARP-III trial provided sufficient evidence about the safety of Sacubitril/Valsartan in patients with moderate-to-severe CKD, particularly if they also had HFrEF, given that it was not associated with worsening renal function or hyperkalemia [33].

In agreement with RCTs, Sacubitril/Valsartan showed a good safety profile also in our population. No cases of symptomatic hypotension were reported in our study. Despite a slight, but not clinically significant increase in the serum potassium levels, only one patient, on concomitant treatment with MRA, exhibited hyperkalemia at T12. Moreover, baseline eGFR did not affect the trend of BP and serum potassium. Regarding pharmacokinetics of a drug mainly excreted in the kidney, there is no evidence of a significant impact of renal impairment on the exposure of Sacubitril/Valsartan [34]. Therefore, the safety of this drug is expected to be maintained even in patients with CKD, as actually observed in HFrEF patients with mild-to-moderate renal dysfunction in RCTs [7] as well as in our study. However, it is important to underline that older patients and patients with CKD mostly took lower dosages of Sacubitril/Valsartan in our real-life clinical practice, as well as in previous reports [22], although the benefits of this new drug appeared to be maintained even at lower dosages [35] and even by adopting a condensed titration regimen [36]. Moreover, real-world data confirmed that Sacubitril/Valsartan was still beneficial in reducing HF hospitalization even in older patients with more comorbidities treated with lower dosages [23]. Finally, a very recent RCT, as secondary end-point, found that Sacubitril/Valsartan was able to improve outcomes even in the acute decompensated HF, without worsening of renal function [37].

### Pathophysiological considerations

The NPs system counteracts the RAAS by lowering BP and exerting multiple beneficial effects on cardio-renal system [38, 39]. The exact mechanism through which Sacubitril/Valsartan preserves eGFR is still unclear. In addition to the cardiac benefits, NPs positively affect distant target organs (vessels, kidney, adipose tissue) and metabolism [5, 40, 41]. The kidney, together with the adipose tissue, is the organ where NP receptors are mainly expressed. In our study, the positive effects of Sacubitril/Valsartan on eGFR were not due only to hemodynamic changes. In fact, together with the eGFR increase, no clinical changes in LVEF and diuretic therapy were reported and there was also a decrease in BP values. Changes in renal function might be related to the direct effect of the pharmacological inhibition of NPs degradation combined with the RAAS blockade on kidney. Neprilysin is mostly expressed in the brush border of proximal renal tubular cells. Its inhibition leads to increased NPs levels, which have been shown to exert protective renal effects in laboratory and clinical settings [42–44]. In experimental models, increased NPs activity had direct effects on proximal tubular reabsorption of sodium and proteins, tubuloglomerular feedback and renal fibrosis [29], exerting direct antioxidant, anti-inflammatory and antifibrotic activities [38, 45]. The glomerular hemodynamics play a central role in the action of NPs on renal function. In stable HFrEF, the reduced kidney perfusion, due to the cardiac systolic dysfunction, leads to the hyperactivation of RAAS that increases the intraglomerular pressure by a predominant vasoconstriction of the efferent arteriole, aiming at maintaining GFR [46]. The inhibition of RAAS prevents the angiotensin II-mediated vasoconstriction of the efferent arteriole, leading to a decrease in intraglomerular pressure and consequently in GFR, that becomes dependent on systemic BP [46]. Therefore, any treatment-induced BP reduction may lead to an increase in serum creatinine levels [47]. The dual inhibition of neprilysin and RAAS by Sacubitril/Valsartan leads to an increase in GFR, despite the reduction in systemic BP and kidney perfusion pressure likely by a preferential vasodilation of the afferent arteriole [46]. However, the increase in intraglomerular pressure, coupled with a possible direct effect of NPs on the glomerular barrier, could explain the increased albuminuria associated with this new drug [48, 49], even though it is not coupled with a deterioration in renal function. It may be a result of the acute intrarenal hemodynamic effects of neprilysin inhibition that tends to stabilize after few weeks of treatment [24]. These mechanisms could affect the preservation of the residual renal function in the long term [50]. Indeed, the long-term renal effects of Sacubitril/Valsartan have been not yet fully elucidated. High-quality studies on the evaluation of the long-term effects of Sacubitril/Valsartan on glomerular perfusion, filtration and sieving function in larger cohorts of patients are needed [46].

### Study limits

To the best of our knowledge, this is the first study focused on the effects of Sacubitril/Valsartan on renal function, following HFrEF patients for 12 months in a real-life clinical context. However, this study has some limitations that have to be pointed out. Although the enrolled patients had a wide age range (22.2% of patients aged ≥ 75 years), the study suffered from the small sample size. As reported in the discussion, some previous RCT found a relationship between Sacubitril/Valsartan and proteinuria. However, data regarding UACR were not available in this study, as well as comprehensive data regarding changes in all echocardiographic parameters. In our analyses, we took into account age, sex, BP and LVEF as major confounding factors. Although no significant changes in other cardiovascular drug therapies were reported, we could not exclude their possible interference on our findings.

## Conclusion

The improved eGFR in real-life HFrEF patients treated with Sacubitril/Valsartan, despite lower BP, represents an important confirmation outside the peculiar world of RCT. This, together with the improved LVEF, may facilitate longer, event-free survivals with lower re-hospitalizations in this population, even in the presence of renal impairment. The mechanisms behind this effect of Sacubitril/Valsartan on renal function are still not fully understood, but it is likely that NPs facilitation has a key role in the context of type-1 angiotensin receptor (AT1) antagonism. Further research is required to elucidate the long-term renal outcomes of this innovative drug class.
